# Diversity study of *Beauveria bassiana* species for finding the most virulent strain to manage *Bemisia tabaci* in cotton

**DOI:** 10.1007/s00253-024-13188-1

**Published:** 2024-06-06

**Authors:** Satish Kumar Sain, Sandhya Kranthi, Keshav Raj Kranthi, Dilip Monga, Debashis Paul, Yenumula G. Prasad

**Affiliations:** 1 ICAR-Central Institute for Cotton Research, Regional Station, Sirsa, Haryana India; 2 ICAR-Central Institute for Cotton Research, Nagpur, Maharashtra India; 3 International Cotton Advisory Committee, Washington, DC USA

**Keywords:** *Beauveria bassiana*, Bioefficacy index, Diversity, Entomopathogens, PCA, SED

## Abstract

**Abstract:**

*Beauveria bassiana* (Bal.-Criv.) is an important entomopathogenic fungus being used for the management of various agricultural pests worldwide. However, all strains of *B. bassiana* may not be effective against whitefly, *Bemisia tabaci*, or other pests, and strains show diversity in their growth, sporulation, virulence features, and overall bioefficacy. Thus, to select the most effective strain, a comprehensive way needs to be devised. We studied the diversity among the 102 strains of *B. bassiana* isolated from 19 insect species based on their physiological features, virulence, and molecular phylogeny, to identify promising ones for the management of *B. tabaci*. Strains showed diversity in mycelial growth, conidial production, and their virulence against B. tabaci nymphs. The highest nymphal mortality (2nd and 3rd instar) was recorded with MTCC-4511 (95.1%), MTCC-6289 (93.8%), and MTCC-4565 (89.9%) at a concentration of 1 × 10^6^ conidia ml^−1^ under polyhouse conditions. The highest bioefficacy index (BI) was in MTCC-4511 (78.3%), MTCC-4565 (68.2%), and MTCC-4543 (62.1%). MTCC-4511, MTCC-4565, and MTCC-4543 clustered with positive loading of eigenvalues for the first two principal components and the cluster analysis also corresponded well with PCA (principal component analysis) (nymphal mortality and BI). The molecular phylogeny could not draw any distinct relationship between physiological features, the virulence of *B. bassiana* strains with the host and location. The BI, PCA, and square Euclidean distance cluster were found the most useful tools for selecting potential entomopathogenic strains. The selected strains could be utilized for the management of the *B. tabaci* nymphal population in the field through the development of effective formulations.

**Key points:**

• *102 B. bassiana strains showed diversity in growth and virulence against B. tabaci.*

• *Bioefficacy index, PCA, and SED group are efficient tools for selecting potential strains.*

• *MTCC-4511, 4565, and 4543 chosen as the most virulent strains to kill whitefly nymphs.*

**Supplementary information:**

The online version contains supplementary material available at 10.1007/s00253-024-13188-1.

## Introduction

Biopesticides have received global attention in recent decades as a safer pest control strategy, either alone or as a component of integrated pest management (IPM) programs. More than 750 fungal species in over 100 fungal genera have been reported as insect pathogens (St Leger and Wang 2010). Entomopathogenic fungi (EPF), with the advantages of non-resistance and non-contamination, are considered a better alternative for whitefly and other pest control than chemicals (Mascarin et al. 2013; Sain et al. 2021, 2022). Even though there are over 20 EPF effective against whitefly, *Bemisia tabaci* (Gennadius), very few bioinsecticides are commercialized for whitefly control. Species such as *Beauveria bassiana, Metarhizium anisopliae, Cordyceps fumosorosea* (synonym: *Isaria fumosoroseus*), *Aschersonia* spp., and *Akanthomyces lecanii* are the most potential entomopathogens of *B. tabaci* (Sain et al. 2019a; 2019b; Zhang et al. 2019). Among these, EPF *B. bassiana* is a generalist insect pathogen able to infect nearly 1000 insect species, and described as the most destructive to whitefly nymphs and adults and is known to cause white muscadine disease (Sain et al. 2019b; 2022). In the past, most EPF were isolated from various ecosystems, and only a few of them were studied for their virulence potential against the target pests including whiteflies. Limited commercial products of EPF are available and provide successful management of insect pests in fields around the world because their selection from few isolates provides a limited scope of finding highly virulent EPF isolates (Imoulan et al. 2017; Inglis et al. 2001; Meyling and Eilenberg 2007; Sain et al. 2019b; 2021; Vega et al. 2009).

In the past, researchers have studied a limited set of EPF and selected the best one for further bioinsecticide development against specific pests. Only a few numbers of *B. bassiana* strains and species are found highly virulent toward *B. tabaci*, and they differ greatly in terms of virulence mechanism. Furthermore, there is no comparison of the molecular, physiological, or pathogenicity characteristics of EPF strains to those of *B. tabaci*. If a large number of EPF with various hosts and locations are screened for their bioefficacy against the target insect pests, the likelihood of discovering the most virulent EPF with improved bioefficacy may be higher. Native strains of EPF from distinct hosts or locations provide higher control of a few indigenous pests as they are fairly more adapted to their surroundings (Faria et al. 2010; Zayed 2003). The nuclear ribosomal repeat unit (5.8S rRNA) and internal transcribed spacer (ITS1 and ITS2) are most widely used for identification and diversity examination of EPF (Arnold 2007; Sabbahi et al. 2009; Shin et al. 2010). Recent studies have revealed that whitefly nymphs at the second-instar stage are more susceptible to EPF than adults (Cuthbertson et al. 2005a, b; Sain et al. 2022; Santiago-Álvarez et al. 2006).

Taking into account the substantial information available, we targeted nymphs to identify highly virulent EPF because this is the most important aspect of controlling whitefly nymphs which ultimately affect the population of adults. The bioefficacy of 102 *B. bassiana* strains collected from six different insect-pest host orders and six states in India was evaluated against whitefly nymphs, Asia II-1. Physiological features, virulence potential, and bioefficacy index of *B. bassiana* to control *B. tabaci* were compared with their molecular diversity using the ITS regions of the nuclear ribosomal repeat unit.

## Materials and methods

### Source of *B. bassiana* strains

**Fig. 1:**
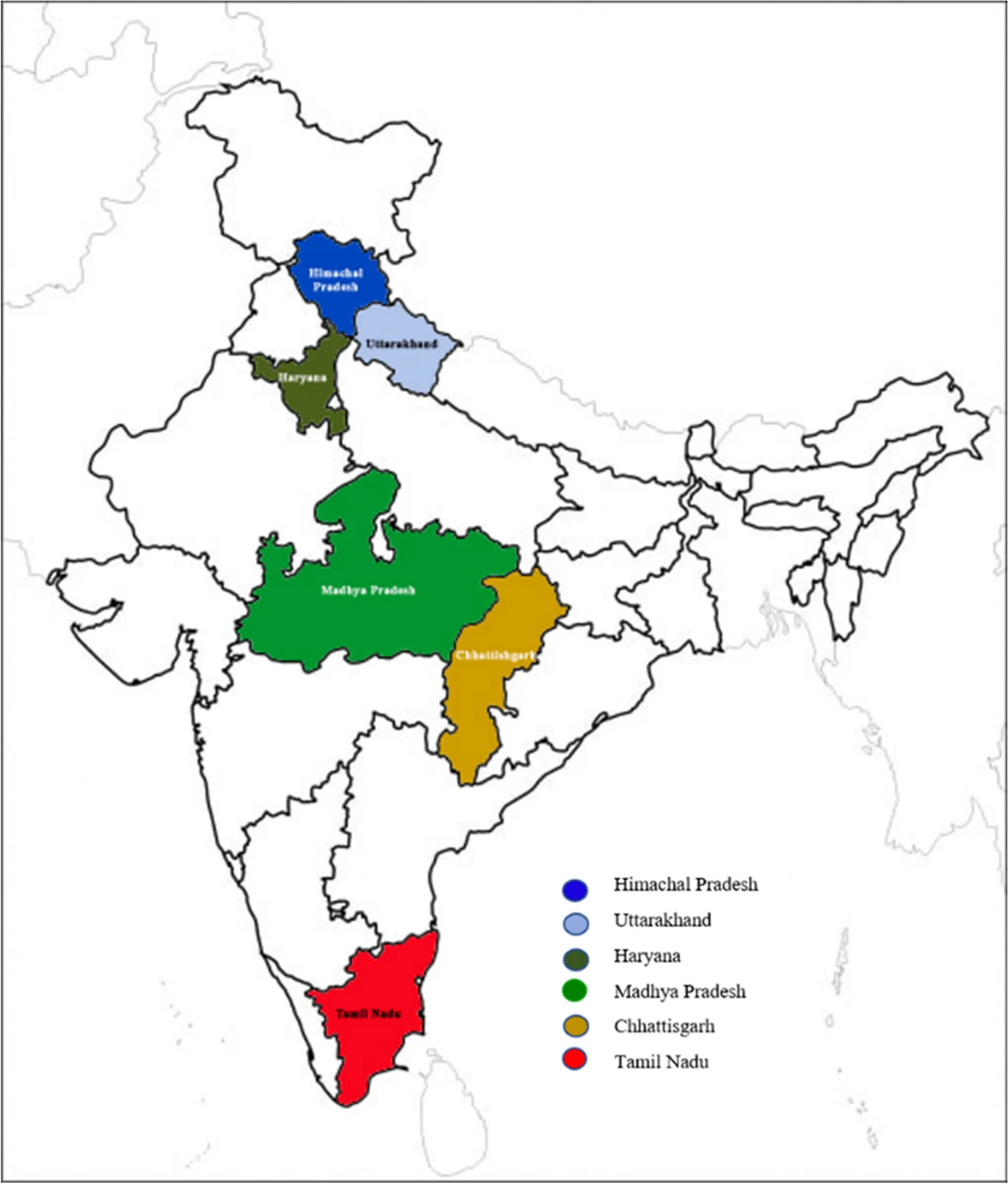
Geographic distribution map of entomopathogenic fungal species *Beauveria bassiana* strains from six different states of India

The 102 strains of entomopathogenic fungi *B. bassiana* originally isolated from different host cadavers of the orders *Coleoptera* (12), *Diptera* (2), *Hemiptera* (3), *Hymenoptera* (5), *Lepidoptera* (68), *Orthoptera* (3), and soil and plant parts (9) from the six states of India including 77 from Madhya Pradesh, 16 from Chhattisgarh, 4 from Tamil Nadu, three from Uttarakhand, and one each from Haryana and Himachal Pradesh were collected from the Microbial Type Culture Collection (MTCC), Chandigarh, India, and used in the study for diversity analysis (Supplementary Table S1, Fig. 1). The purified *B. bassiana* strains isolated from the host cadavers, soil and plant samples belonging to *Eutectona machaeralis*, *Plusia orichalcea*, *Hyblaea puera*, and *Bombyx mori* of the order *Lepidoptera*, *Pieris rapae*, *Drosicha mangiferae*, *Ideoscopus clypeali*, and *Mocis columbia* of the order *Hemiptera*, *Oryctes rhinocerous*, beetle and grub of the order *Coleoptera*, Black ant of the order *Hymenoptera*, fly of the order *Diptera*, grass hopper of the order *Orthoptera*, infested larva, soil, etc. All the cultures of *B. bassiana* strains were sub-cultured and maintained on Sabouraud Dextrose Agar (Hi-Media, Mumbai, India) with yeast extract (0.2%) and streptomycin sulfate (20 μg l^−1^) (SDYA) in sterile Petri plates and incubated in biochemical oxygen demand (BOD) incubator at 28 ± 2 °C in the dark for 10–15 days. *B. bassiana* cultures were stored in a refrigerator at 4 °C on SDYA Petri plates for bioassay tests.

### Diversity in mycelial growth and conidial production

Growth and production of mycelia and conidia by each *B. bassiana* strain were assessed with slight modifications in the method described by Cheng et al. (2016). Mycelial disks (5 mm in diameter) taken from 10-day-old cultures were placed in the middle of the SDYA Petri plates (90 mm dia, Hi-Media). Then, the Petri plates were covered with parafilm strips and incubated at 28 ± 2 ^○^C, 80 ± 5% relative humidity (RH) and 12-h photoperiod for 7–10 days. The mycelial growth was observed daily (diameter in mm) with a ruler scale and the final observation was recorded 7 days after inoculation (DAI). The two-sided perpendicular diameters of each colony were measured and the average of each treatment/colony was used for analysis and comparison. Conidial concentration was recorded at 7 DAI for each strain, separately for comparison with other strains. For this, a 5-mm 10-day-old culture disk was cut using a sterile cork borer and dropped into the test tube containing distilled water. Aqueous Tween 80 (PEG-80 sorbitan monolaurate, Hi-Media) (0.1 ml of 0.02%) was added to it and the spores were carefully scraped with the help of a small paintbrush. Later, to achieve a homogeneous suspension, the conidial suspension was vortexed to break up clumps or chains of conidia. The sterile-distilled water was used to make the basic initial solution of 10 ml for each EPF isolate. A serial dilution procedure was followed to make other concentrations from 10^–3^ to 10^–6^ using an improved Neubauer hemocytometer. The per unit area spore number was recorded, analyzed, and expressed as conidia/ml. Three replications (5 mm culture disk) for each treatment/strain were maintained separately and the experiment was repeated thrice.

### Diversity in bioefficacy potential

*B. bassiana* strains were incubated on SDYA in sterilized Petri plates (90 mm) at 28 ± 2 °C in darkness before the initiation of the bioassay experiment. The sterile 0.01% (v/v) Tween 80 solution was poured into 10-day-old culture plates of each strain and stirred with a glass rod. This suspension was used for making the conidial stock solution. The stock suspension was further vortexed and filtered using fourfold layers of nylon cheesecloth. The concentration of conidial filtrate (10 ml) of individual strain was standardized using an improved Neubauer hemocytometer and compound microscope at × 400 magnification. Bioefficacy assays were performed using the conidial concentration of 1 × 10^6^ conidia ml^−1^ with 0.01% (v/v) surfactant (Tween 80, Hi-Media). Prior to every bioassay, the conidial viability of each conidial suspension was confirmed using the germination test on the SDYA medium (Hi-Media) at 28 ± 2 °C for a period of 24 h (Faria et al. 2010; Sain et al. 2022). This germination test was repeated for each stock suspension, and after 24-h incubation, the viability of conidia (≥ 95% germination) was ascertained prior to the bioassay test (Goettel and Inglis 1997).

In this study, the *B. tabaci* population was reared and maintained in a polyhouse (500 m^2^) on potted plants of upland cotton variety HS-6. The comparative bioefficacy of each *B. bassiana* strain was assessed by following the nymphal mortality bioassay test under polyhouse conditions (Sain et al. 2019a). Adult whiteflies (Asia-II-1) *B. tabaci* population for this study were collected from cotton field trials of ICAR-Central Institute for Cotton Research-Regional Station (29°32′36.1″N 75°02′18.8″ E) and were identified based on the *mtCOI* gene sequence analysis. The sequence (MN329161, MN329162, and MN329163) showed 94–95% nt identity with the whitefly cryptic species Asia II-1- [China: Zhejiang] sequence (AJ867557) and clustered in Asia II-1 phylogenetic group (Biswas et al. 2020; Sain et al. 2022). After the release of the ~ 50 whitefly adults on potted HS-6 plants, the population reached 50–60 whiteflies per leaf within one month. Then, for bioassay experiments, pest-free 30-day-old potted plants (5–6 fully expanded primary leaves of HS-6 variety) were placed near the infested plants for 24 h for egg laying inside the polyhouse. Whitefly adults were gently removed from the potted plants with the help of a hand-held low-pressure air pump 24 h post-egg-laying, and plants were shifted to another polyhouse. Within the next 10 days, the nymphs reached 0.30–0.44 mm in length and 0.18–0.36 mm in width (the 2nd instar) (Mascarin et al. 2013; Quintela 2004; Sain et al. 2019b; 2022). Controlled temperature (30 ± 2 °C), relative humidity (RH) (75 ± 2%), and a diurnal day/night cycle (16/8 h) were maintained in the polyhouse. Later, 10-day post-egg-laying the observations were recorded by marking a circle around each nymph on the abaxial surface of the leaf by a waterproof marker (40–50 nymphs per leaf).

*B. bassiana* culture suspension (10^7^ conidia ml^−1^) was spray-inoculated on marked nymphs on the leaves of cotton plants. Tween 80 solution (0.01%) was sprayed on the control plants. Three replications were maintained for each treatment/strain and each replication contained three potted cotton plants with three tagged leaves on each (a total of 27 leaves for each treatment). The nymphal mortality was recorded prior to spray treatments and later at 3-, 5-, and 7-day post-spray treatment (DAI) using a hand magnifying lens (× 20). The bioassay experiment was repeated twice for all the treatments. While recording the whitefly nymphal mortality data, live nymphs showed an opaque or shining greenish-white color, and brown eyes or honeydew droplets on the excretions, while dead nymphs were yellowish-brown mat color with shriveled body shapes (Sain et al. 2021; 2022). A uniform population of nymphs was used throughout this experiment. Corrected mortality % = [(mortality % in treatment − mortality % in control) / (100 − mortality % in control)] × 100. The virulence of all the strains against whitefly nymphs was expressed as corrected nymphal mortality.

The bioefficacy index (BI) has been described as an indicator for choosing the best EPF strain to apply in the fields (Sain et al. 2019b; 2022). To compare the diversity, the BI for each strain was calculated following the formula, BI = 0.37 × MG + 0.13 × SP + 0.50 × MO (Sain et al. 2021, 2022), where MG = mycelial growth (mm) at 10 DAI, SP = sporulation (1 × 10^8^ conidia/ml) at 10 DAI, and MO = nymphal mortality at 7-day post-spray treatment. The BI was calculated using the 15%, 35%, and 50% weightage for mycelial growth, sporulation, and nymphal mortality, respectively (Sain et al. 2019b). Furthermore, for comparison and grouping of the *B. bassiana* strains, the BI reactions were categorized into six groups based on BI and the Ward’s method of clustering and the squared Euclidean distance as HV = highly virulent (≥ 60); GV = good virulent (50–59), V = virulent (40–49), MV = moderately virulent (30–39), LV = low virulent (20–29), and PV = poor virulent (≤ 19).

### Statistical analysis of data

A replicated completely randomized design (CRD) was used to carry out all experiments. The data from the bioassay experiments conducted in the laboratory, polyhouse, were statistically analyzed by means of using analysis of variance (ANOVA) of CRD. Corrected mortality data obtained using Schneider-Orelli’s formula were subjected to the variance analysis (Puntener 1981). Furthermore, the BI was considered for comparison of bioefficacy among *B. bassiana* strains. The test dates of experiments were considered experimental blocks; however, due to potential restriction errors, the block interactions were not tested (Sokal and Rohlf 1995).

Following Shapiro–Wilk and Brown–Forsythe tests and diagnostic residual plots, datasets were checked for normality and homoscedasticity (Jaeger 2008). The data in percentage values were arcsine transformed before statistical analysis. Online computer software OP Stats was used to calculate the Least Significant Difference Test (LSD) at *P* ≤ 0.05 and to compare treatment effects (Sheoran et al. 1998). Critical differences among the EPF strains regarding mortality rates, mycelia growth, and conidia production were calculated at *P* ≤ 0.05 level of significance by ANOVA and subsequently for comparison of treatment by LSD multiple comparison test. All statistical analyses were conducted using the “Statistical Analysis System” (SAS) Packages 2020 by SAS Institute, Cary, NC, USA.

To identify the best strains of *B. bassiana* considering six measured response virulence factors/variables (categories), a principal component analysis (PCA) was used. Virulence factors include mycelial growth and conidial production on media and BI including whitefly nymphal mortality. The SAS 2020 program was used to perform PCA, and extract the independent variables and provide a direct measurement of the total variance through the PCA biplot, score plot, and eigenvectors. The principal axis method and varimax (orthogonal) rotation were used to extract the components and to identify the observed variables, respectively. The PCA was accomplished with standardized data (mean = 0, variance = 1); the variables were measured in different scales and variables that confirmed high loading for each component retained in the analysis. The eigenvalues of the PCA are characterized as a measure of their associated variance, and the influence of each observable variable in these components was assumed by the loadings (Lê et al. 2008). Pearson’s correlation coefficient was projected to test the correlations between individual components and variables. Each component was defined only by the variables that are significant at a significance threshold of *P* ≤ 0.15. Principal components that explained at least 70% of the total variance were used to draw biplot graphs and further used in the interpretation of results.

Dendrograms for the bioefficacy of different strains were prepared based on the BI data of each strain. All the strains were categorized under 6 different clusters using Ward’s method of clustering and the squared Euclidean distance was inferred to estimate the genetic distance among the strains. Additionally, a cluster analysis was executed with the unweighted pair-group of the arithmetic mean (average UPGMA) technique to compute the Euclidean distances of divergence (Everitt and Hothorn 2011). This technique provided the number of clusters that emerged among the *B. bassiana* strains based on the meaningful detected variables resulting from PCA. The highest similarities between strains were scored as having the shortest distances.

Internal transcribed spacer (ITS)-1 and ITS-2 sequences of 102 *B. bassiana* strains retrieved from GenBank were accumulated and aligned with the help of the web interface of the multiple sequence alignment program of MEGA X version 10.1 (Molecular Evolutionary Genetics Analysis) (Kumar et al. 2018). The adjustment in the alignments was done so as to allow maximum alignment and minimize gaps. A molecular phylogenetic tree was constructed using the MEGA X version 10.1. software. The evolutionary distances tree was figured out using the neighbor-joining method (Saitou and Nei 1987). Phylogenetic trees were developed using the heuristic search option including 1000 random sequence additions. The resulting max trees were unlimited, zero-length branches were collapsed, and all trees of multiple parsimonious were saved. The optimal tree was inferred with the sum of branch length = 24.52394125. The percentage value of replicate trees in which the associated taxa clustered together in the bootstrap test (500 replicates) is shown next to the branches (Felsenstein 1985). The evolutionary distances were subtracted with the Maximum Composite Likelihood method (Tamura and Nei 2004) which is mentioned in the units of the number of base substitutions per site. This investigation intricated 102 nucleotide sequences and the codon positions comprised were 1st + 2nd + 3rd + noncoding. Ambiguous positions were detached for each sequence pair using the pairwise removal option. A total of 590 positions were recorded in the final dataset. The ITS-based phylogeny dendrogram was compared with each strain of *B. bassiana*, their location, host, and BI to compare their diversity and relationship with virulence.

## Results

All 102 strains of *B. bassiana* demonstrated a varying degree of diversity at cultural, and virulence to cause whitefly nymphal mortality, bioefficacy index (BI), and molecular levels.

### Cultural and physiological diversity

**Fig. 2:**
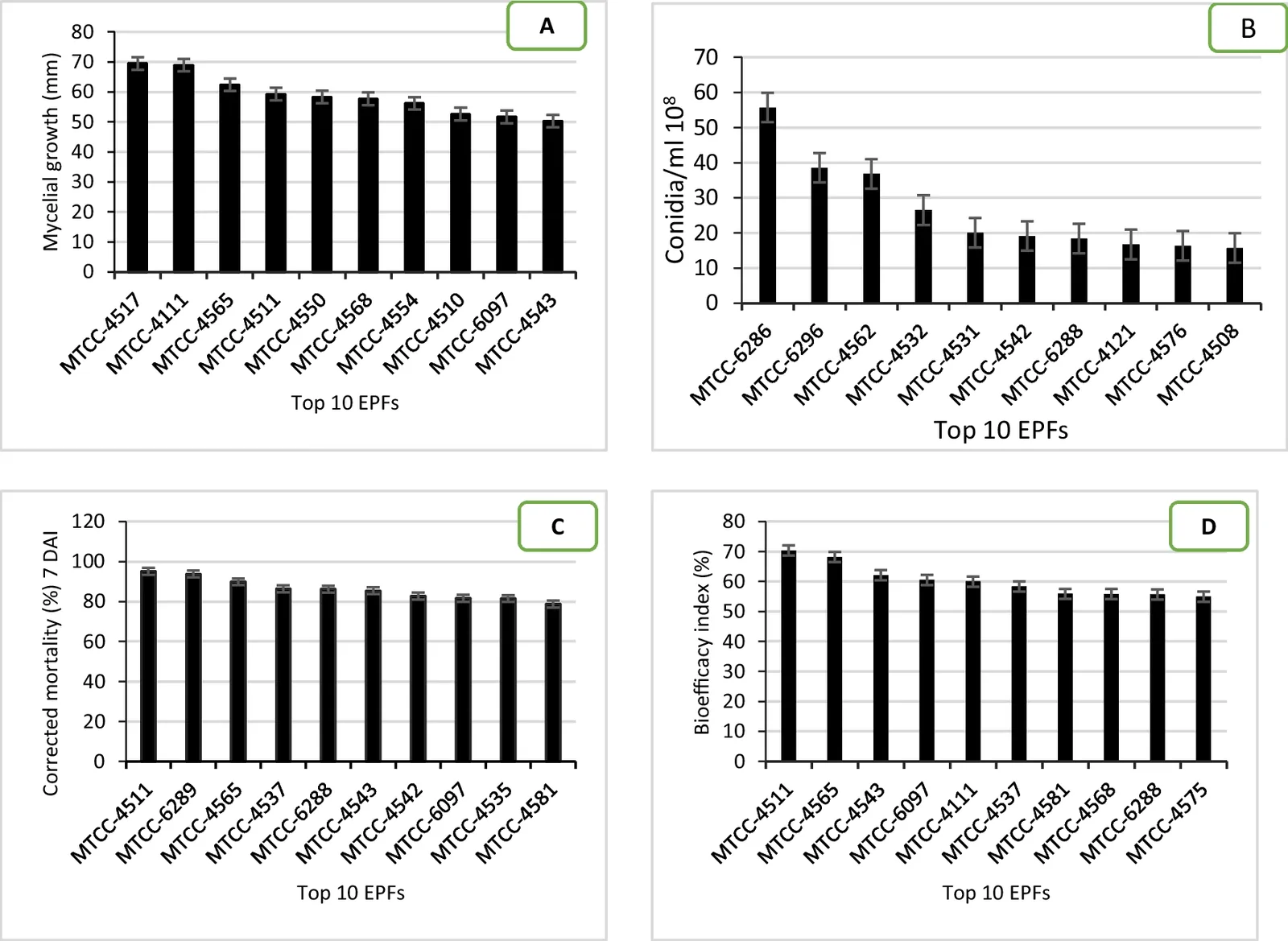
Top ten EPFs observed with a higher mycelial growth rate at seven days post-inoculation (**A**), conidial production rate at ten days post-inoculation (**B**), percent corrected whitefly nymphal mortality rates of (2nd and 3rd instar) (means ± SE) at 7 days after inoculation under polyhouse (**C**) and bioefficacy (%) of EPFs (**D**). Error bars represent the standard deviation for three replicates (SE ± 4.08 for data **A**; SE ± 0.89 for **B**; SE ± 3.97 for data **C** and SE ± 2.79 for data **D**)

The results of the laboratory tests revealed that all the strains of *B. bassiana* showed a very wide range of diversity in their mycelial growth and conidial production. The average mycelial growth at 7-day post-incubation (DAI) and conidial production at 10 DAI ranged from 8.4 to 69.5 mm and from 0.3 to 55.7 × 10^8^ conidia/ml, respectively (Supplementary Table S2). Among 102 *B. bassiana* strains, the top ten strains showed a mycelial growth diameter in the range from 50.3 to 69.5 mm with the highest in MTCC-4517 (69.5 mm) followed by MTCC-4111 (68.9 mm) and MTCC-4565 (62.4 mm) (Fig. 2a). The conidia production ranged from 0.3 to 55.7 (at 10^8^ conidia/mg/ml) with the highest in MTCC-6286 (55.7) followed by MTCC-4562 (36.8) and MTCC-4532 (26.5). These strains differ statistically significantly from one another for mycelial growth and conidia production (*P* ≤ 0.05) (Fig. 2b, Table 1).

**Table 1:** Overall bioefficacy index percent (means ± SE) of entomopathogenic fungal isolates

Accession number^@^	Mycelial growth (cm^2^)	Spore × 10^8^/ml	Abbotts corrected mortality (%)^$^^,^^&#^			Bioefficacy index (%)^#^
			3 DAI*	5 DAI	7 DAI	
MTCC-4511	59.3	6.5	75.0 (60.0)^ab^	88.6(70.3)^a^	95.1(77.2)^a^	70.3(57.0)^a^
MTCC-4565	62.4	1.5	57.2 (49.1)^c^	76.6(61.1)^b^	89.9(71.5)^ab^	68.2(55.7)^ab^
MTCC-4543	50.3	5.9	67.4(55.2)^bc^	80.0(63.4)^ab^	85.4(67.5)^b^	62.1(52.0)^abc^
MTCC-6097	51.7	4.4	23.6(29.1)^d^	64.4(53.4)^c^	81.7(64.7)^b^	60.5(51.1)^bc^
MTCC-4111	68.9	0.4	32.9(35.0)^d^	64.8(53.6)^c^	68.9(56.1)^c^	60.0(50.8)^bc^
MTCC-4537	37.7	9.9	83.6(66.1)^ab^	83.7(66.2)^a^	86.4(68.4)^ab^	58.4(49.8)^c^
MTCC-4581	44.2	1.3	60.0 (58.3)^b^	74.8 (59.9)^b^	78.8 (62.6)^b^	55.9 (48.4)^c^
MTCC-4568	57.7	0.4	58.8 (50.1)^bc^	64.5 (53.4)^c^	68.7 (56.0)^c^	55.8 (48.3)^cd^
MTCC-6288	27.6	18.4	82.0(64.9)^ab^	82.6 (65.3)^a^	86.2 (68.2)^ab^	55.7 (48.3)^cd^
MTCC-4575	49.0	12.8	56.8 (48.9)^bc^	69.8 (56.7)^b^	70.3 (57.0)^c^	55.0 (48.3)^cd^
MTCC-6289	16.8	7.5	83.2 (65.8)^a^	87.1 (69.0)^a^	93.8 (75.6)^ab^	54.1 (47.4)^cde^
MTCC-4542	16.0	19.1	70.4 (57.0)^b^	72.9 (58.6)^bc^	82.8 (65.5)^b^	49.8 (44.9)^de^
MTCC-4535	10.5	2.5	76.9 (61.3)^ab^	80.0 (63.4)^a^	81.5 (64.5) ^b^	45.0 (42.1)^e^
MTCC-4549	44.2	2.3	48.3 (48.9)^bc^	62.8 (52.4)^c^	73.5 (59.0)^c^	53.4 (46.9)^e^
LSD at 0.05	8.03	1.61	7.47	7.20	7.84	5.46
SE(m)	2.89	0.58	2.68	2.58	2.80	1.97
SE(d)	4.08	0.89	3.68	3.65	3.97	2.79
CV	15.73	15.74	11.79	9.71	9.61	9.47

^$^Values are means of three replications (three plants with three leaves each) for each of the EPF isolates tested under polyhouse conditions

^@^Top 15 EPF isolates tested using a spore concentration of 1 × 10^7^ conidia ml^−1^

^&^Figures in parenthesis are arcsign transformed values

^#^For corrected nymphal mortality means within a column followed by the same upper-case letter and means within a row followed by the same lower-case letter do not differ significantly (LSD, *P* ≤ 0.05)

^*^DAI, days after inoculation

### Diversity in bioefficacy potential

A significant difference between the strains of *B. bassiana* to cause whitefly nymphal mortality (2nd and 3rd instar) at 3–, 5–, and 7–DAI was observed (*P* ≤ 0.05) under polyhouse conduction. The nymphal mortality at 3-, 5-, and 7-DAI ranged from 0.9 to 83.6, 3.5 to 88.6, and 4.7 to 98.1%, respectively (Supplementary Table S2). The top ten *B. bassiana* strains caused whitefly nymphal mortality between 78.8 and 95.1% with the highest mortality by MTCC-4511 (95.1%) followed by MTCC-6289 (93.8%) and MTCC-4565 (89.9%) with the concentration of 1 × 10^6^ conidia/l^−1^ at 7-DAI under 80.3–68.4% RH and 33.7–26.7 °C max. and min. temperature (Fig. 1;* P* ≤ 0.05) (Fig. 2c). The comparison of nymphal mortality showed highly significant statistical differences between strains (*P* ≤ 0.05). Thus, for selecting the most suitable EPF strain based on its potential bioefficacy, the inclusive BI was compared. The trend of the BI data results was neither similar with fungal growth character nor with nymphal mortality. However, a significant difference (*P* ≤ 0.05) between the bioefficacy of EPF strains was detected. The overall BI ranged from 9.5 to 70.3% and the top ten strains resulted in a BI above 55.0%. The highest BI was recorded with MTCC-4511 (70.3%) followed by MTCC-4565 (68.2%) and MTCC-4543 (62.1%) (Fig. 2d, Table 1).

### Principal component analysis and clustering with square Euclidean distance

**Fig. 3:**
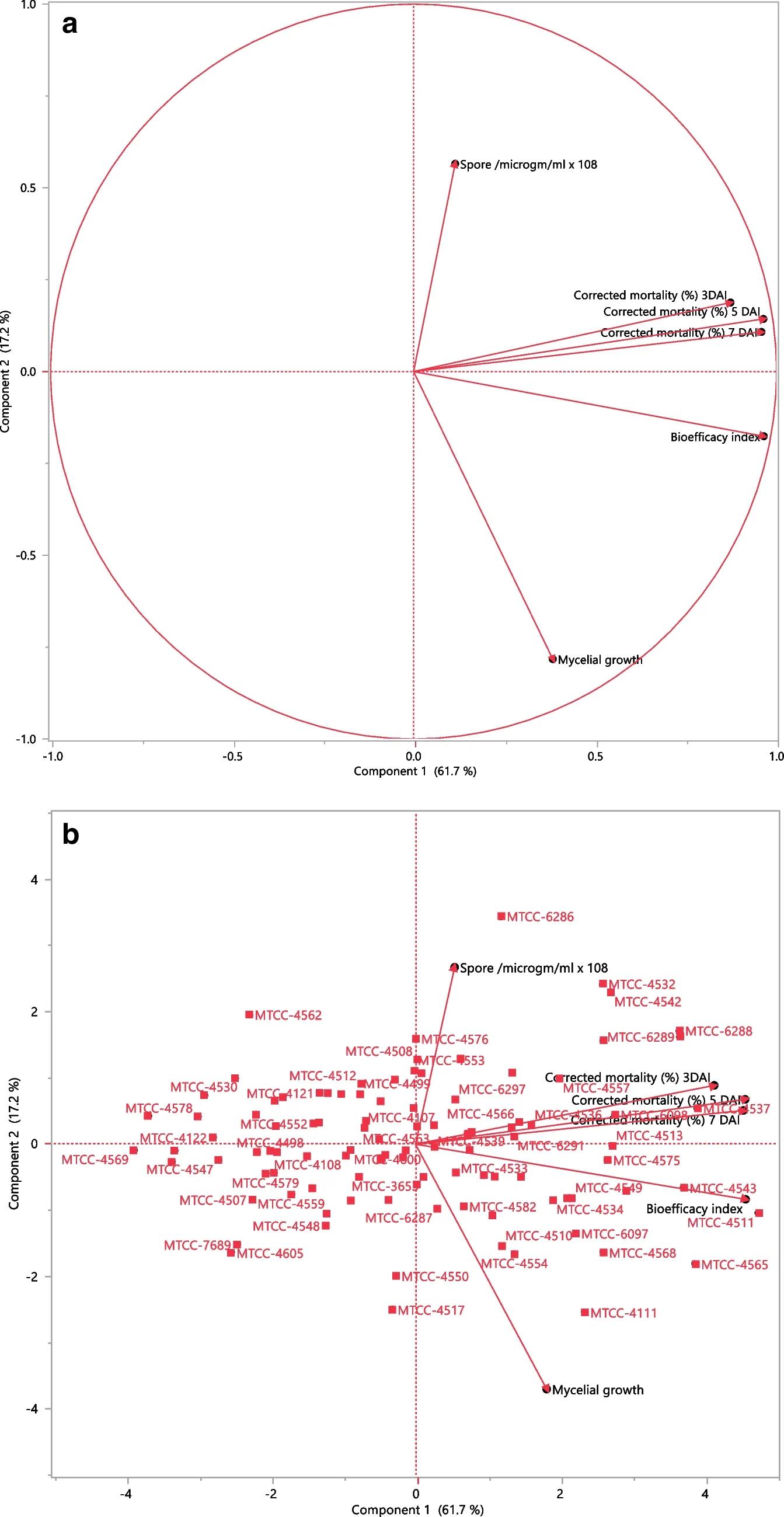
Biplot score graph pictures from PCA between components 1 and 2 which explained 78.8% of the total variance in the observed variables including different growth and virulence factors (*n* = 6) on bioefficacy of entomopathogenic fungal strains (*n* = 102). **A** Variables’ graph considering only active variables, in which the length of the arrows indicates the magnitude of its respective variable. **B** Individuals’ graph showing the scores for the EPF strains, according to the first two principal components. Component 1 comprises the variables “corrected mortality (%) 3 DAI,” “corrected mortality (%) 5 DAI,” “corrected mortality (%) 7 DAI” and “bioefficacy index,” while component 2 is designated by “mycelial growth (cm)” and “Spore ml × 10^8^”

The PCA elaborated the extent of variability among the EPF strains which was useful in finding linear relationships between the important detected variables, i.e., the efficacy of strains in mycelial growth, conidial production, nymphal mortality, and BI. Components 1 and 2 explained 78.85% of the total variance and displayed eigenvalues were greater than 1.03 (Table 2). Hence, the biplot graph drawn with only two components of the dataset which comprises six variables. The involvement of each variable on each axis in the biplot graph was measured proportionally through the length of the arrows. Together first two PCA components (PC1 and PC2) comprised significant variables at *P* < 0.15, following Pearson’s correlation method. For the first principal component, variables “nymphal mortality at 3 DAI,” “nymphal mortality at 5 DAI,” “nymphal mortality at 7 DAI” and “BI” were found the most important and in turn, resulted in the maximal amount of total variance (61.2%) (Fig. 3a). The PC2 described 17.2% of the total variance and comprises the variables “mycelial growth,” and “conidial production” (Fig. 3a).

**Table 2:**
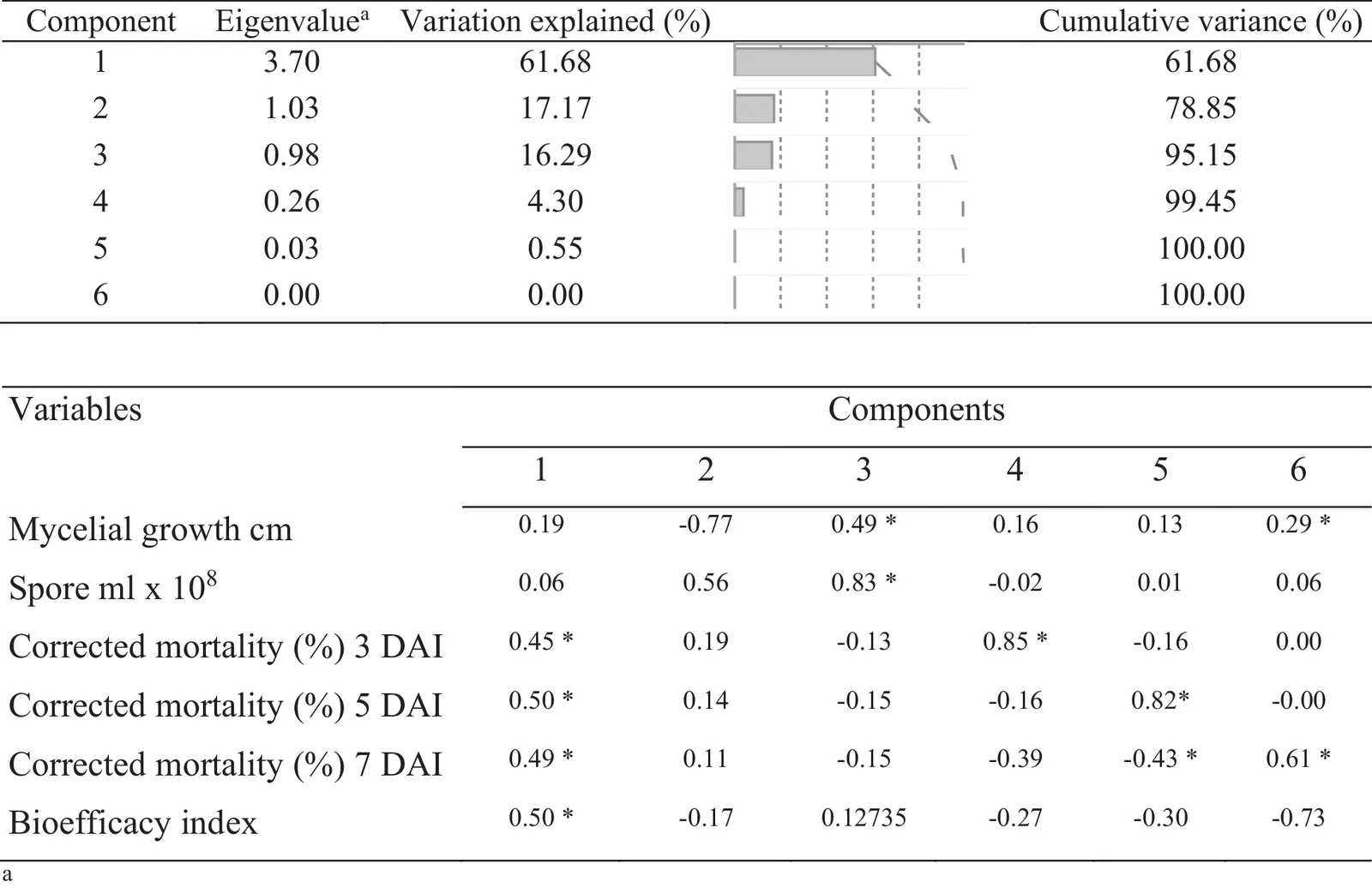
Principal component analysis with eigenvalues and variance explained for each principal component

*Attribute loading for eigenvectors (rotated factor pattern) and correlations between variables and the two meaningful components retained^*^

^a^Eigenvectors are presented for components 1 to 6. There were just two principal components retained which account for 78.85% of the total variance in the data set. Correlations (*r*, Pearson’s method) between variables and the principal components with a significance threshold at *P* ≤ 0.15 are shown. Since components 1 and 2 comprised all the meaningful variables, the other components were no longer important to build the biplot graph

The biplot for individual variables (Fig. 3b) explained strains with high virulence to nymphs and high nymphal mortality. Three out of 102 *B. bassiana* strains, MTCC-4511, MTCC-4565, and MTCC-4543, resulted in higher whitefly nymphal mortality and were associated with other variables positively, demonstrating high BI with greater conidial production and virulence against nymphs. However, MTCC-4530, MTCC-4578, and MTCC-4569 were with low BI (least virulent under the bioefficacy category in Table 3), MTCC-6286 with higher conidial production characters and MTCC-4111 with higher mycelial growth. Nevertheless, MTCC-4511, MTCC-4565, and MTCC-4543 showed high virulence against nymphs, as they showed higher BI.

**Table 3:**
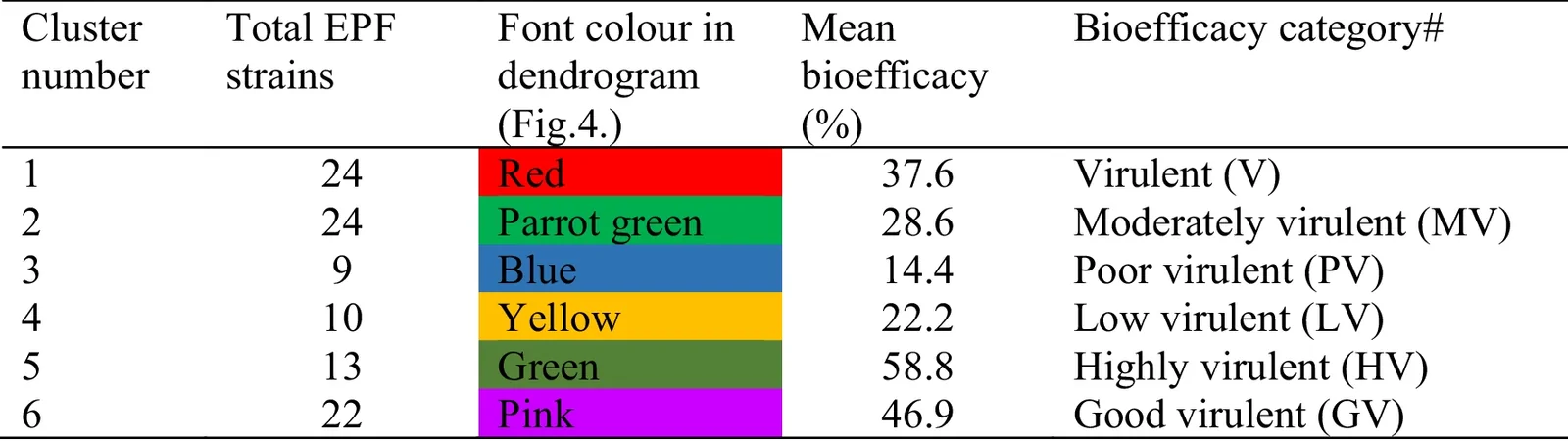
Categorization of *Beauveria bassiana* strains under different clusters/ virulence categories based on bioefficacy index values (%) and the Ward’s method of clustering and the squared Euclidean distance

^#^The reaction categorized as *HV*, highly virulent (53.4–70.3); *GV*, good virulent (50–59); *V*, virulent (40–49), *MV*, moderately virulent (30–39); *LV*, low virulent (20–29); *PV*, poor virulent (below 19)

**Fig. 4:**
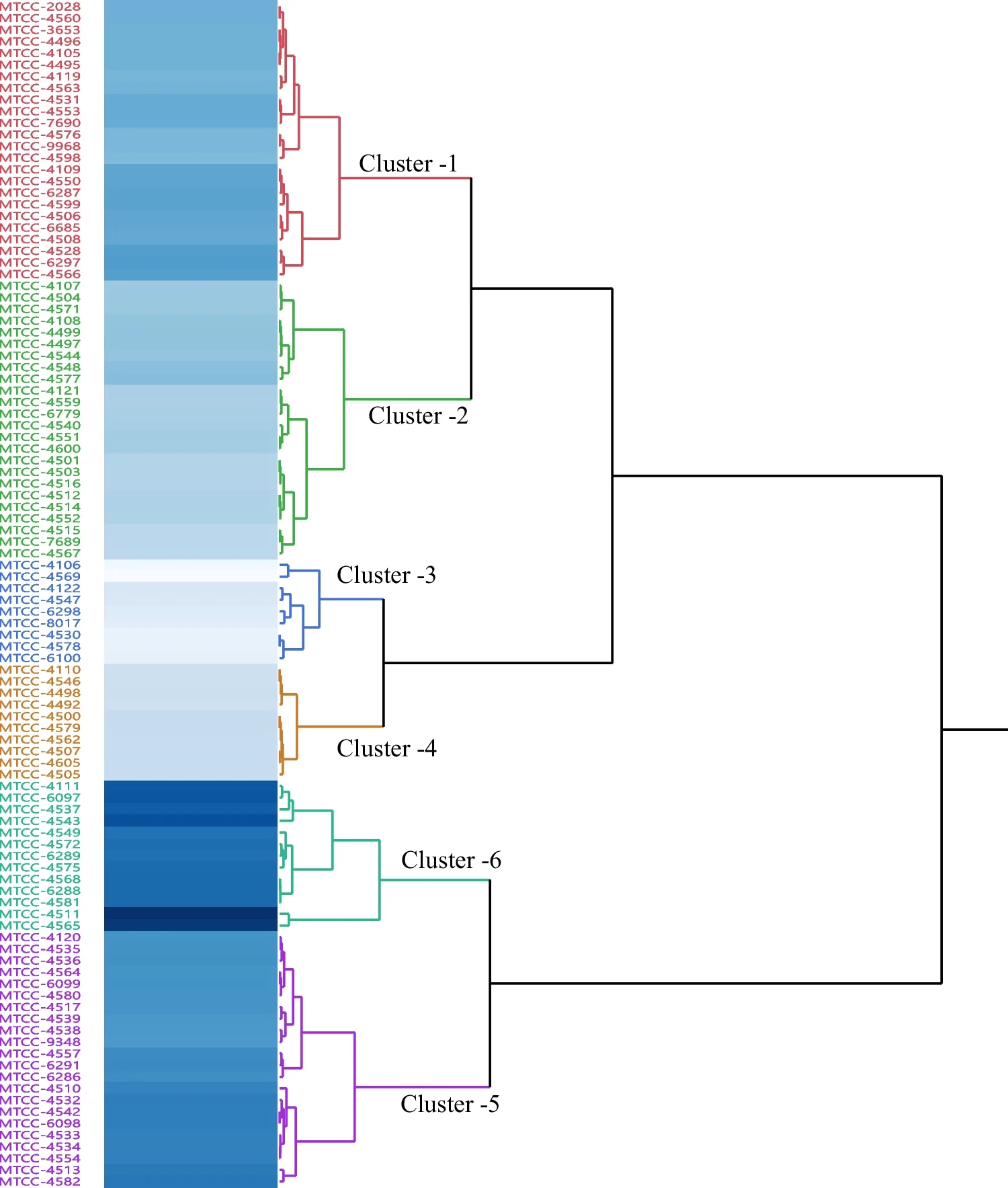
Clustering and the squared Euclidean distance dendrogram for bioefficacy index of different *Beauveria bassiana* strains

The dendrogram for the BI of 102 different strains was categorized into six clusters using the Ward’s method of clustering, and the squared Euclidean distance was inferred to estimate the bioefficacy distance among the strains **(**Fig. 3). Based on the BI of the strains, 13 strains fall under cluster 5 of highly virulent (53.4–70.3%), 22 under cluster 6 good virulent (42.7–52%), 24 under cluster 1 virulent (33.8–42.1%), 24 under cluster 2 moderately virulent (24.7–33%), 10 under cluster 4 low virulent (20.8–23.2%), and 9 were cluster 3 poor virulent (9.8–18%) (Fig. 3). The average BI clusters in decreasing order were observed to be 58.8% (cluster 5), 46.9% (cluster 6), 37.6% (cluster 1), 28.6% (cluster 2), 22.2% (cluster 4), and 14.4% (cluster 3) (Table 3). The cluster analysis proposed six separate groups with alike profiles (Fig. 4). The shortest distances in the dendrogram represented the most closely related performances. Consequently, one cluster (number 6) comprised the 13 strains of *B. bassiana* with the MTCC-4511 and IMTCC-4565 in one single subcluster. Other strains of *B. bassiana* were clustered in separate subclusters of cluster 6. Hence, the *B. bassiana* strains with high BI values show the potential for use in IPM of cotton whiteflies in the fields.

### ITS-based phylogenetic diversity based on location, EPF host, and bioefficacy index (BI)

**Fig. 5:**
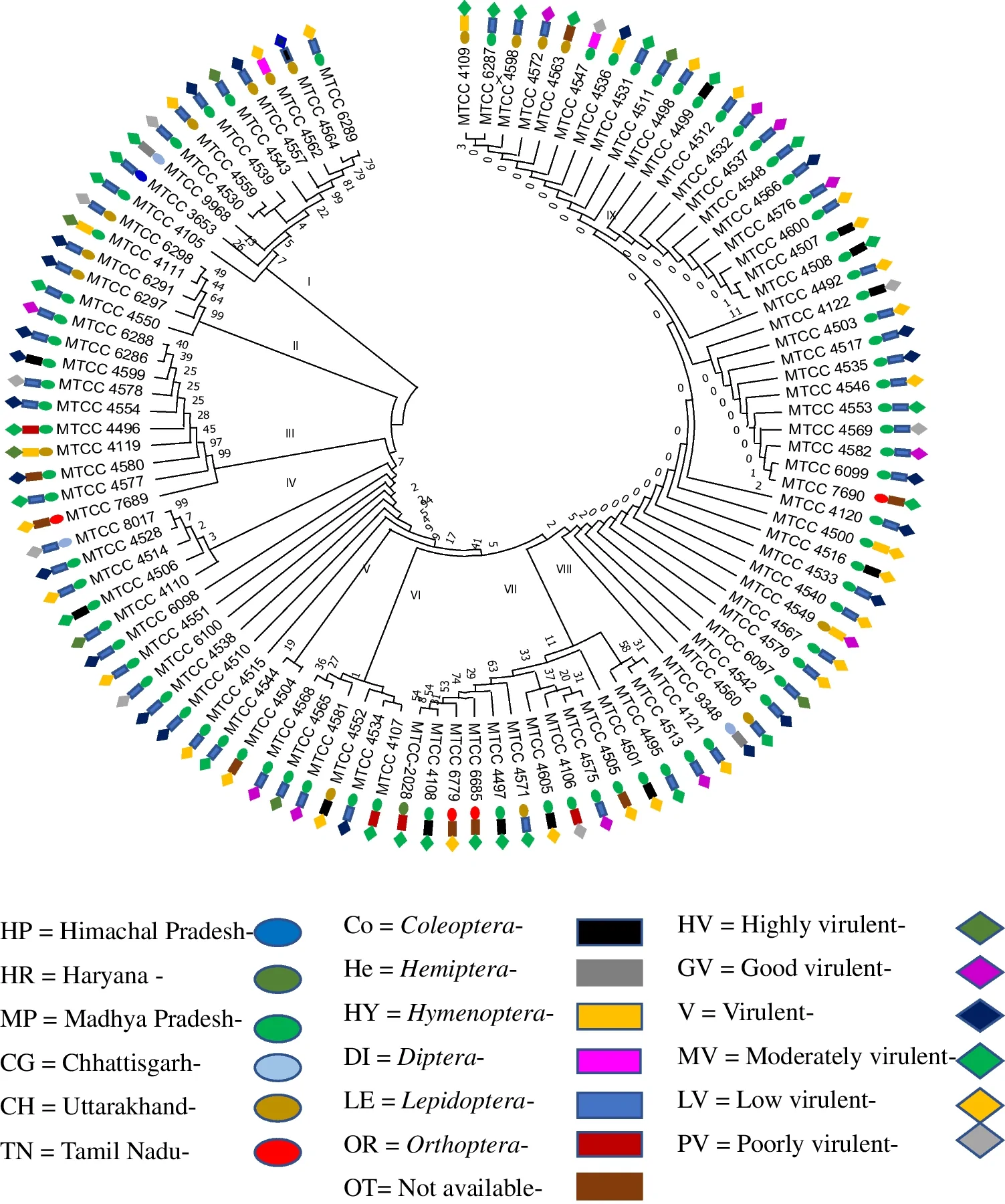
Phylogenetic analysis of *B. bassiana* strains based on rDNA ITS sequences by neighbor-joining method and their respective location, host insect, and bioefficacy index. Figure legends and boxes with different colors and shapes for location, EPF strain host cadavers, and bioefficacy index are explained above

For the physiology of the strains of *B. bassiana* based on the ITS sequence dataset, the location, host, and BI of 102 sequences were included. The ITS sequences were further subjected to construct a dendrogram by the neighbor-joining method using the Clustal-W and MEGA X version 10.1 (Fig. 5). The percentage value of replicate trees showing the associated taxa clustered together in the bootstrap test (500 replicates) is indicated next to the tree branches. The evolutionary distances using the maximum composite likelihood method were computed and depicted in the units of the number of base substitutions per site. A total of 102 nucleotide sequences and codon positions including 1st + 2nd + 3rd + noncoding were analyzed. Ambiguous positions of each sequence pair (pairwise deletion option) were removed and a total of 590 positions were retained in the final dataset. Phylogenetic analyses based on gene sequences consisting of 102 *B. bassiana* strains resolved most lineages of *B. bassiana* in separate terminal branches which exposed a similar tree and clustering topology. Evolutionary analyses conducted using MEGA X revealed that 102 sequences of *B. bassiana* strains clustered in 10 distinct large genetic groups/clades (Fig. 5). Clade-I included 11 strains of *B. bassiana* MTCC-6289, 4564, 4562, 4557, 4543, 4539, 4559, 4530, 9968, 3653, and 4105 which were isolated from the insect cadavers belonging to 4 insect orders namely *Coleoptera*, *Diptera*, *Hemiptera*, and *Lepidoptera* collected from different locations in 4 states of India including Chhattisgarh, Uttarakhand, Himachal Pradesh, Madhya Pradesh, and fall under the all BIclusters 1 to 6 (poor virulent to highly virulent) (Fig. 5). Similarly, Clade-II included 5 strains MTCC-4550, 6298, 6297, 6291, and 4111 isolated from the insect cadavers belonging to the order *Hemiptera*, *Lepidoptera* collected from different locations in 2 states of India including Chhattisgarh, Madhya Pradesh, and fall under the all BI clusters 6, 1, and 2 (good virulent, virulent, and moderately virulent, respectively). Also, Clade-III included 10 strains of *B. bassiana* MTCC-6288, 6286, 4599, 4578, 4554, 4496, 4119, 4580, 4577, and 7689 which were isolated from the insect cadavers belonging to 3 insect orders namely *Coleoptera*, *Lepidoptera*, and *Orthoptera* collected from different locations in 3 states of India including Chhattisgarh, Madhya Pradesh, and fall under all BI clusters 1 to 6 (poor virulent to highly virulent). Clade IV included 5 strains of *B. bassiana* MTCC-8017, 4528, 4514,4506, and 4110 were isolated from different insect orders and locations. The most virulent EPF strains are MTCC-4511 (clade X), MTCC-4565 (clade VI), and MTCC-4543 (clade I). The ITS sequence investigation of all the *B. bassiana* strains described an average of 95–100% similarity of sequences that necessitates the conspecific nature of the strains and their host cadavers from which the strains were isolated irrespective of geographic regions. The phylogenetic tree of EPF strains also depicted the diversity of *B. bassiana* within species complex (in each clade) irrespective of location, host insects, and BI and falls into 10 different clades and without any similarity.

## Discussion

### Cultural and physiological diversity

The *B. bassiana* species depict species diversity richness and are wide-ranging in nature as soilborne necrotrophic fungi. This species complex is a cosmopolitan group of arthropod-pathogenic fungi that have prodigious potential for various insect pest management. Investigation on biopesticides has demonstrated that entomopathogenic fungal bioinsecticides are effective and eco-friendly alternatives to chemicals for controlling several economically important insect-pests. Several EPF are being exploited for *B. tabaci* management and the virulence of EPF is considered the major factor for their selection (Borisade and Mahan 2015; Cuthbertson et al. 2005a, b; Sain et al. 2019b; 2022; Quesada-Moraga et al. 2006). Important criteria referred to for selecting entomopathogens for the development of commercial formulation should be the potential for fungal growth and production of spores and high virulence against the target pest (Sain et al. 2019b; 2022). Considering this criterion, considerable information is available on the efficiency and potential use of *B. bassiana* for managing numerous insect species, we studied the diversity of 102 strains of *B. bassiana* collected from 19 different locations in the six states of India, host cadavers of 19 insect species belonging to six orders *Coleoptera, Diptera, Hemiptera, Hymenoptera*, *Lepidoptera*, and *Orthoptera*, and plant, soil samples (Supplementary Table 1). We further evaluated different fungal attributes, using the BI, PCA, and clustering to identify the most virulent isolates for managing whitefly Asia-II-1. We reported the diversity of *B. bassiana* strains at physiological, virulence against whitefly nymphs, BI, and molecular level.

It has been well documented that in the EPF life cycle, spores/conidia as the first and last stage and mycelial growth play a vital role in determining the virulence potential of an EPF. Hence, studies on proliferation and virulence bioassay against the target pest must be conducted prior to the selection of any EPF for further deployment in field conditions (Sain et al. 2022). The production potential of spores by an EPF helps cause epizootic in the target insect pest in the fields (Joseph et al. 2010), and furthermore, the spore inoculum affects the insect’s behavior in terms of killing the host (Joseph et al. 2010; Leão et al. 2015). Thus, for EPF perpetuation, causation of infection, and proliferation, factors like the growth of mycelia and production of conidia become important. Moreover, the reproduction mechanism (mycelial growth and sporulation) is governed by fungal genetic, hormonal, nutritional, and environmental features. However, the fungal species’ survival depends on the ability to produce viable spores subsequently after mycelial growth under diverse ecological conditions and the physical and nutritional requirements which are more stringent than those required for growth of mycelia (Moore 1996). Hence, for effective pest management, these factors along with virulence (insect mortality) become essential and integral parts (Sain et al. 2019a; 2022).

During the study, we evaluated the production of mycelial growth and conidia from 102 native strains of *B. bassiana* in the laboratory on SDYA which include glucose as a carbon source and yeast extract as a nitrogen source. In our work, strains of *B. bassiana* showed significant variability in mycelial growth at seven DAI ranging from 8.4 to 69.5 mm diameter at 28 ± 2 ^○^C, 80 ± 5% RH, and 12 h photoperiod on SDYA (Fig. 2a). Similarly, the conidial production at 10 DAI ranged from 0.3 to 55.7 × 10^8^ conidia/mg/ml at seven DAI (Supplementary Table S2). The mycelial growth was highest in MTCC-4517, MTCC-4111, and MTCC-4565 while conidia production was highest in MTCC-6286, MTCC-4532, and MTCC-4562. These strains differ significantly from one another (*P* ≤ 0.05) both for mycelial growth and conidial production. We have not observed any correlation between mycelial growth, sporulation, and location and host of the *B. bassiana* strain (Fig. 2b, Table 2). It has been stated that EPF may vary in their ability to utilize the carbon and nitrogen sources for reproduction since growth and spore production favor mono-saccharides like fructose or glucose. Some sources of nitrogen are observed to enhance mycelial growth but do not favor spore production, but nitrogen sources like asparagine and the ammonium compounds inhibit the growth and spore production due to the ammonia accumulation during the growth (Moore 1996). The nitrogen source like yeast extract is more effective in boosting the mycelial growth and spore production, though, at low concentrations (0.25 to 0.50%) the growth of mycelia remains below the optimum and the spore production remains low at very high concentrations (1.75 to 2%) (Devi 1994). Several experiments have elucidated the effect of nutrition and proportions of carbon/nitrogen (C/N) ratio on mycelial growth and spore production for *M. anisopliae*, *Paecilomyces lilacinus*, *B. bassiana*, and *Hirsutella rhossiliensis* to choose the best suitable components, optimal concentrations best strains (Gao and Liu 2010; James 2001; Liu and Chen 2002; Safavi et al. 2007). These authors observed that the growth of mycelia and the spore production differed between diverse species and strains. However, we have evaluated only single-growth media (SDYA) and found significant variation in the mycelial growth and sporulation among the *B. bassiana* strains. Similarly, James (2001) has also observed the difference in mycelial growth and conidia production while evaluating a few strains of *B. bassiana*. Previous studies indicate variation in in vivo spore production within fungal species, which might disturb horizontal transmission and infection of the pests in the fields. It has been revealed that *I. fumosorosea* can cause epizootics in whitefly populations under best-suited weather conditions, mostly in cloudy and wet weather (Lacey et al. 2015). Sporulation on cadavers of whiteflies is very much dependent on high relative humidity nonetheless varies with temperature range, fungal strain, host species, and stage, and time of incubation (Sosa-Gómez and Alves 2000).

We have also detected proportionally greater sporulation responses in some strains compared to other strains of *B. bassiana* which could be an added advantage for horizontal transmission in the field. The production of aerial spores of EPF strains on solid substrata varies among species and strains. Seeds of rice are found to be the most commonly utilized medium for the solid-state fermentation of EPF (Li et al. 2011) and a higher conidial yield of *I. fumosorosea* CG1228 is recorded. On the other hand, Mascarin et al. (2010) have observed maximum yield of *I. fumosorosea* and *B. bassiana* in a biphasic fermentation process on whole rice, and preboiled rice grains were found conducive for higher conidia production in solid-state fermentation due to its better nutritional and physical properties. However, to the best of our information, there is no report with a comparison of spore production and mycelial growth between a large number of *B. bassiana* strains on the same nutrient source. The alteration in the morphology and nutrient components can expose the connection between conidial production and mycelial growth, and provide a systems-level understanding of the cell. Also, there may be a difference between the strains in their genetics and molecular regulation which regulates the appearance of morphological features (Papagianni 2004).

### Diversity in bioefficacy potential

In this study, all the strains of *B. bassiana* were found to be virulent against *B. tabaci* nymphs (ASIA-II-1) and showed a statistically significant variation in the nymphal mortality (2nd and 3rd instar) with the concentration of 1 × 10^6^ conidia/ml at 7 DAI in the max. and min. temperature 33.7–26.7 °C and RH 80.3–68.4% (Fig. 1; *P* < 0.05). The mortality ranged from 4.7 to 95.1% with the top ten strains causing whitefly nymphal mortality > 78.8% (Fig. 2c, Table 1). Our results corroborate the *B. bassiana* strains association with *B. tabaci*, which causes variable levels of mortality in the *B. tabaci* nymphs (Borisade and Mahan 2015; Lacey et al. 2015; Sain et al. 2019b). Some studies sustenance the potential of *B. bassiana* and *I. fumosorosea* for the mortality in whiteflies (Cabanillas and Jones 2009; Cuthbertson et al. 2005a, b; Sain et al. 2022; Vicentini et al. 2001; Wraight et al. 1998). *B. bassiana* caused whitefly nymphal mortality ranging from 78.2 to 95.1% at 1 × 10^6^ conidia ml^−1^ under polyhouse conditions (Faria and Wraight 2001; Sain et al. 2019b; 2022). Similarly, Mascarin et al. (2013) recorded 71–81% nymphal mortality within 8 days by *B. bassiana* and *I. fumosorosea*, while the median lethal time (LT_50_) values ranged from 3 to 4 days with 10^–7^ conidia ml^−1^. Lacey et al. (2008) stated that for 50% nymphal mortality in *B. tabaci* biotype B, 50–150 spores/mm^2^ were essential. Moreover, factors other than isolate, like experimental design, stage of the host, environmental conditions, and surfactant, can also affect the results of diverse bioassays (Liu and Stansly 2000; Srinivasan et al. 2008). However, in the present study, the 102 strains of *B. bassiana* caused 4.7 to 95.1% mortality with a similar dose (1 × 10^6^ conidia ml^−1^). This clearly shows the genetic variability within the species of *B. bassiana*. Moreover, in the present study, the *B. tabaci* populations belong to the whitefly cryptic species Asia II 1 based on sequences of the partial *mtCOI* gene and supported by the earlier studies (Ashfaq et al. 2014; Ellango et al. 2015; Naveen et al. 2017; Pan et al. 2018).

As per this present study, the best strains of *B. bassiana* based on virulence to *B. tabaci* nymphs were MTCC-4511, MTCC-6289, and MTCC-4565. These best strains can be utilized in the biocontrol of whiteflies through an inundation approach. Additionally, these *B. bassiana* strains can easily be mass multiplied on solid substrates and are compatible with different types of pesticide formulation (Faria and Wraight 2001; Jackson et al. 2010; Mascarin et al. 2010; Sain et al. 2019a; 2022). The bioefficacy of EPF has been specified to rely on their virulence factor and propagation (spore production, mycelia growth, and metabolite production) and good physical properties (adequate conidia > 1 million ml^–1^, long-term shelf life, long-lasting activity) are much required for the synthesizing an effective formulation and to deliver effective control of target pests consistently in the field (Mascarin et al. 2010; Jackson et al. 2010; Sain et al. 2018, 2022). Adequate spore production by an EPF and their germination ability are the most important components that are needed for adequate shelf-life, proliferation, and virulence. These essential components are integral parts of the formulation for successful management of target pests. Hence, it is conducted that a comprehensive study to find out the bioefficacy potential of each strain of *B. bassiana* is needed*.* Our study showed variability of strains for all three factors and a single strain usually has not performed all three activities consistently. The three parameters (i) the nymphal morality, (ii) mycelial growth, and (iii) spore production was measured to calculate the overall BI. The top ten strains resulted in a BI above 55.0% and strains MTCC-4511, MTCC-4565, and MTCC-4543 turned out highly bio-efficacious and promising candidates for biological control of *B. tabaci* ASIA-II-1 and will be designated for development of bioinsecticides. This indicates that strains with relatively better bioefficacy would play a vital role in the reduction of both the nymphal and adult populations as well as their survival and further transmission in cotton fields. The studies conducted in past corroborate our hypothesis of remaining conidia activated from dead nymphs in the field which would further help horizontal transmission of EPF and cause epizootic (Gindin et al. 2000; Negasi et al. 1998; Lacey et al. 1999; Ramos et al. 2000). Additional studies are ongoing to develop effective formulation to optimize the efficacy and persistence of selected EPF strains for adoption in integrated whitefly management under field conditions.

### Diversity grouping using PCA and clustering with SED

PCA and cluster analysis proposed that nymph mortality and BI (mycelial growth, conidial production, and mortality) response were significant and relevant variables for strain variability and virulence screening. The biplot graphs of PCA highlighted the magnitude of diversity within *B. bassiana* strains in causing nymphal mortality and their physiological characteristic, which is elucidated by the clusters spread along the two PC axes. The diagram demarcated the features that are dispersed along two PC axes and stresses the extent of phenotypic variation explicated by the clusters. The features that positively correlated and executed best in the respective variables are on the upper right side of the quadrant (Fig. 3). The *B. bassiana* strains and the components those were existing together and in close proximity to one another confirmed better virulence. In our investigation, PCA analysis was able to identify nymphal mortality, and BI as the important factors for their selection and discussing their variability. Among 102 strains, MTCC-4511, MTCC-4565, and MTCC-4543 grouped with positive loading of eigenvalues for the first two PC (Table 1, Fig. 3). The other variables such as mycelial growth and conidial production alone have not affected the virulence of strains on whitefly nymphs. Based on our results, *B. bassiana* strain MTCC-4511 was well identified as a promising strain for biocontrol control of *B. tabaci* Asia-II-1 through bioinsecticide formulation, while MTCC-4565 is also promising. The cluster analysis also corresponded well with the PCA results. Furthermore, clustering and the squared Euclidean distance dendrogram for the BI of *B. bassiana* strains divided them into six groups. These strains fall in one subgroup of cluster 6 of the most efficacious strains and are shown to have similarities with the PCA sub-component (Table 3, Fig. 4).

### Diversity relationship of ITS-based phylogenetic with location, EPF host, and bioefficacy index

Furthermore, the molecular phylogeny based on ITS sequences was developed by using the ITS region to explore the genetic diversity within the *B. bassiana* strains. The phylogenetic tree highlighted the evolutionary relationship within *B. bassiana* strains with a wide range of their virulence against whitefly nymphs, collected from different hosts in diverse geographical regions of India. One hundred two strains studied in the present study were clustered into ten evolutionary lineages (6 major and 4 minor) of *B. bassiana* strains. In all the lineages, variation in EPF strain virulence was very much prominent and there was no similarity or correlation with the strain’s virulence, their host, and/or geographical location. This suggests that the *B. bassiana* strains isolated from different hosts, and geographical regions may differ ominously in their virulence (Fig. 5). Similar to our study, the virulence of Brazilian strains of *B. bassiana* at different temperatures and potential of horizontal transmission on *Cosmopolites sordidus* adults were studied by Lopes et al. (2011). They have not observed any relation between molecular groups and the virulence of *B. bassiana* against *C. sordidus*. Meyling et al. (2009) and Agrawal et al. (2014a) used DNA microsatellite markers to examine the infra-specific diversity of 102 isolates of *B. bassiana* sensu stricto from India which indicated the occurrence of highly polymorphic, randomly scattered populations of *B. bassiana* with diverse host range and without host-specificity. They not found any apparent correlation among the isolates, host, and geographical location that revealed high allelic diversity among *B. bassiana* isolates. However, based on reduced datasets, they could observe region-wise clusters of the populations, which indicates the bigger role of geographical/ecological locations rather than host-pathogen interactions for *B. bassiana* isolates from India.

In the previous studies, Agrawal et al. (2014b) have done ITS-based phylogenetic analysis of 111 *Beauveria* spp. isolates that grouped within the *Beauveria* clade, while 14 *Beauveria*-like isolates showed phylogenetic affinities with *Isaria* and *Tolypocladium* clades. They also analyzed multi-gene phylogenetics including the partial gene sequence data of *Bloc*, *EF1α*, *RPB1*, and *RPB2* showing different clusters of the different species. However, Bidochka et al. (2002) have proposed that the relationships of *B. bassiana* isolates with its host may be accidental, it is primarily habitat-associated rather than host-associated. In more recent studies, Wang et al. (2002) evaluated 15 species and their phylogenetic positions according to phylogenetic inferences based on six loci (nrSSU, nrLSU, TEF, RPB1, RPB2, and Bloc). They determined the virulence potential of the *B. bassiana* complex and *B. scarabaeidicola* complex on the *B. mori*, *Tenebrio molitor* larvae, and *Protaetia brevitarsis* adults. However, they could not draw any clear-cut relationship between the host and the virulence. The findings of the current study are also in coherence with the earlier findings (Meyling et al. 2009; Wang et al. 2002) that the virulence potential of *B. bassiana* strains is not able to establish relationships among the diverse population with their specific host and particular geographic location. Hence, virulence plays a vital role in managing insect pests, and bioefficacy-centric screening could be useful implications in future biocontrol research for finding the most virulent strains of entomopathogenic fungi.

In conclusion, several EPF strains were selected based on their virulence as the major and applied for the management of *B. tabaci*. However, we have found that strains of *B. bassiana* showed their variability for mycelial growth, conidial production, nymphal mortality, and BI. The virulence and BI do not have any relationship with the host and location of *B. bassiana* strains. PCA and cluster analysis supported our findings that the bioefficacy of each strain has a strong relationship with conidial production and virulence (nymphal mortality). The present study also suggests that the BI (mycelial growth, conidial production, virulence) is a useful tool for finding the potential strain of *B. bassiana* and other EPF foreseeing the probable maximum field efficacy. Furthermore, the results indicate that strains with relatively better bioefficacy would play a vital role in the control of whitefly nymph, adult populations, and CLCuD severity in the field. The selected strains of *B. bassiana* MTCC-4511, MTCC-4565, and MTCC-4543 turned out highly bio-efficacious (≥ 62%) causing nymphal mortality (≥ 85%) and can further be used as promising candidates for inundative biocontrol approach against *B. tabaci* ASIA-II-1 through the development of bioinsecticides.

## Supplementary information

Below is the link to the electronic supplementary material.

Supplementary file1 (PDF 134 KB)

## Data Availability

All data supporting the findings of this study are available within the paper and its Supplementary Information.
