# Supplementary material for: Diversity study of Beauveria bassiana species for finding the most virulent strain to manage Bemisia tabaci in cotton
Source: Appl Microbiol Biotechnol. 2024 Jun 6;108:364. doi: 10.1007/s00253-024-13188-1 (PMC11156744; doi:10.1007/s00253-024-13188-1)
Supplement: Supplementary file 1 [file 253_2024_13188_MOESM1_ESM.pdf]

Applied Microbiology and Biotechnology

Diversity study of *Beauveria bassiana* species for finding the most virulent strain to manage *Bemisia tabaci* in cotton

Satish Kumar Sain[1#], Sandhya Kranthi[3], Keshav Raj Kranthi[3], Dilip Monga[1], Debashis Paul[1] and Yenumula G. Prasad[2]

[1]ICAR-Central Institute for Cotton Research, Regional Station, Sirsa, Haryana, India

[2]ICAR-Central Institute for Cotton Research, Nagpur, Maharashtra, India

[3]International Cotton Advisory Committee, Washington, DC, USA

[#] Corresponding author email: sain.skumar@gmail.com

ORCID ID: 0000-0003-4378-8363

Supplementary Table S1: Hosts, and locations of the evaluated entomopathogenic isolates collected from microbial type culture collection (MTCC)

S.No	Accession number	Host	Host order	Location	State*	Host order
1.	MTCC-2028	NA	NA	IMTECH	HR	OR
2.	MTCC-3653	Oak-tasar silkworm pupae- *Bombyx mori*	*Lepidoptera*	Mandi	HP	LE
3.	MTCC-4105	*Hyblaea puera*	*Lepidoptera*	Mandla	MP	LE
4.	MTCC-4106	Grasshopper	*Orthoptera*	Jabalpur	MP	OR
5.	MTCC-4107	Grasshopper	*Orthoptera*	Jabalpur	MP	OR
6.	MTCC-4108	Crop beetle	*Coleoptera*	Jabalpur	MP	CO
7.	MTCC-4109	Black ant	*Hymenoptera*	Bastar	CG	HY
8.	MTCC-4110	*Eutectona machaeralis*	*Lepidoptera*	Mandla	MP	LE
9.	MTCC-4111	Small black ant	*Hymenoptera*	Jabalpur	MP	HY
10.	MTCC-4119	*Mocis columbia*	*Hemiptera*	Bastar	CG	HE
11.	MTCC-4120	*Pieris rapae*	*Lepidoptera*	Mandla	MP	LE
12.	MTCC-4121	*Pieris rapae*	*Lepidoptera*	Mandla	MP	LE
13.	MTCC-4122	Beetle	*Coleoptera*	Mandla	MP	CO
14.	MTCC-4492	*Pieris rapae*	*Lepidoptera*	Katni	MP	LE
15.	MTCC-4495	*Hyblaea puera*	*Lepidoptera*	Katni	MP	LE
16.	MTCC-4496	Grasshopper	*Orthoptera*	Katni	MP	OR
17.	MTCC-4497	Crop beetle	*Coleoptera*	Katni	MP	CO
18.	MTCC-4498	*Eutectona machaeralis*	*Lepidoptera*	Balaghat	MP	LE
19.	MTCC-4499	Beetle	*Coleoptera*	Balaghat	MP	CO
20.	MTCC-4500	Black ant	*Hymenoptera*	Balaghat	MP	HY
21.	MTCC-4501	Grub	*Coleoptera*	Balaghat	MP	CO
22.	MTCC-4503	*Plusia orichalcea*	*Lepidoptera*	Betul	MP	LE
23.	MTCC-4504	NA	NA	Betul	MP	OT
24.	MTCC-4505	NA	NA	Betul	MP	OT
25.	MTCC-4506	Beetle	*Coleoptera*	Betul	MP	CO
26.	MTCC-4507	Beetle	*Coleoptera*	Betul	MP	CO
27.	MTCC-4508	Beetle	*Coleoptera*	Betul	MP	CO
28.	MTCC-4510	*Eutectona machaeralis*	*Lepidoptera*	Betul	MP	LE
29.	MTCC-4511	*Eutectona machaeralis*	*Lepidoptera*	Betul	MP	LE
30.	MTCC-4512	*Eutectona machaeralis*	*Lepidoptera*	Betul	MP	LE

31.	MTCC-4513	*Hyblaea puera*	*Lepidoptera*	Betul	MP	LE
32.	MTCC-4514	*Hyblaea puera*	*Lepidoptera*	Hoshangabad	MP	LE
33.	MTCC-4515	*Hyblaea puera*	*Lepidoptera*	Hoshangabad	MP	LE
34.	MTCC-4516	Beetle	*Coleoptera*	Betul	MP	CO
35.	MTCC-4517	*Hyblaea puera*	*Lepidoptera*	Betul	MP	LE
36.	MTCC-4528	*Plusia orichalcea*	*Lepidoptera*	Chhindwara	MP	LE
37.	MTCC-4530	*Plusia orichalcea*	*Lepidoptera*	Betul	MP	LE
38.	MTCC-4531	*Plusia orichalcea*	*Lepidoptera*	Betul	MP	LE
39.	MTCC-4532	*Hyblaea puera*	*Lepidoptera*	Hoshangabad	MP	LE
40.	MTCC-4533	*Hyblaea puera*	*Lepidoptera*	Hoshangabad	MP	LE
41.	MTCC-4534	*Hyblaea puera*	*Lepidoptera*	Hoshangabad	MP	LE
42.	MTCC-4535	*Eutectona machaeralis*	*Lepidoptera*	Hoshangabad	MP	LE
43.	MTCC-4536	Black ant	*Hymenoptera*	Hoshangabad	MP	HY
44.	MTCC-4537	*Eutectona machaeralis*	*Lepidoptera*	Hoshangabad	MP	LE
45.	MTCC-4538	*Eutectona machaeralis*	*Lepidoptera*	Hoshangabad	MP	LE
46.	MTCC-4539	*Eutectona machaeralis*	*Lepidoptera*	Hoshangabad	MP	LE
47.	MTCC-4540	*Eutectona machaeralis*	*Lepidoptera*	Hoshangabad	MP	LE
48.	MTCC-4542	*Eutectona machaeralis*	*Lepidoptera*	Chhindwara	MP	LE
49.	MTCC-4543	*Eutectona machaeralis*	*Lepidoptera*	Chhindwara	MP	LE
50.	MTCC-4544	*Eutectona machaeralis*	*Lepidoptera*	Chhindwara	MP	LE
51.	MTCC-4546	*Plusia orichalcea*	*Lepidoptera*	Chhindwara	MP	LE
52.	MTCC-4547	Fruit fly	*Diptera*	Chhindwara	MP	DI
53.	MTCC-4548	*Plusia orichalcea*	*Lepidoptera*	Chhindwara	MP	LE
54.	MTCC-4549	Black ant	*Hymenoptera*	Mandla	CG	HY
55.	MTCC-4550	*Eutectona machaeralis*	*Lepidoptera*	Seoni	MP	LE
56.	MTCC-4551	*Eutectona machaeralis*	*Lepidoptera*	Seoni	MP	LE
57.	MTCC-4552	Beetle	*Coleoptera*	Mandla	CG	CO
58.	MTCC-4553	*Hyblaea puera*	*Lepidoptera*	Hoshangabad	MP	LE
59.	MTCC-4554	*Eutectona machaeralis*	*Lepidoptera*	Seoni	MP	LE
60.	MTCC-4557	*Eutectona machaeralis*	*Lepidoptera*	Mandla	CG	LE
61.	MTCC-4559	*Eutectona machaeralis*	*Lepidoptera*	Mandla	CG	LE
62.	MTCC-4560	*Eutectona machaeralis*	*Lepidoptera*	Mandla	CG	LE
63.	MTCC-4562	Fly	*Diptera*	Mandla	CG	DI

64.	MTCC-4563	Unidentified insect	NA	Mandla	CG	OT
65.	MTCC-4564	Beetle	*Coleoptera*	Mandla	CG	CO
66.	MTCC-4565	*Plusia orichalcea*	*Lepidoptera*	Jabalpur	MP	LE
67.	MTCC-4566	*Plusia orichalcea*	*Lepidoptera*	Jabalpur	MP	LE
68.	MTCC-4567	*Plusia orichalcea*	*Lepidoptera*	Jabalpur	MP	LE
69.	MTCC-4568	*Plusia orichalcea*	*Lepidoptera*	Jabalpur	MP	LE
70.	MTCC-4569	*Plusia orichalcea*	*Lepidoptera*	Jabalpur	MP	LE
71.	MTCC-4571	*Eutectona machaeralis*	*Lepidoptera*	Mandla	CG	LE
72.	MTCC-4572	*Eutectona machaeralis*	*Lepidoptera*	Mandla	CG	LE
73.	MTCC-4575	*Eutectona machaeralis*	*Lepidoptera*	Jabalpur	MP	LE
74.	MTCC-4576	*Eutectona machaeralis*	*Lepidoptera*	Jabalpur	MP	LE
75.	MTCC-4577	*Eutectona machaeralis*	*Lepidoptera*	Jabalpur	MP	LE
76.	MTCC-4578	*Eutectona machaeralis*	*Lepidoptera*	Jabalpur	MP	LE
77.	MTCC-4579	*Eutectona machaeralis*	*Lepidoptera*	Jabalpur	MP	LE
78.	MTCC-4580	Insect	NA	Jabalpur	MP	OT
79.	MTCC-4581	*Plusia orichalcea*	*Lepidoptera*	Jabalpur	MP	LE
80.	MTCC-4582	*Plusia orichalcea*	*Lepidoptera*	Jabalpur	MP	LE
81.	MTCC-4598	*Eutectona machaeralis*	*Lepidoptera*	Bastar	CG	LE
82.	MTCC-4599	Beetle	*Coleoptera*	Shahdol	MP	CO
83.	MTCC-4600	*Plusia orichalcea*	*Lepidoptera*	Shahdol	MP	LE
84.	MTCC-4605	Beetle	*Coleoptera*	Shahdol	MP	CO
85.	MTCC-6097	*Eutectona machaeralis*	*Lepidoptera*	Mandla	MP	LE
86.	MTCC-6098	*Eutectona machaeralis*	*Lepidoptera*	Mandla	MP	LE
87.	MTCC-6099	*Eutectona machaeralis*	*Lepidoptera*	Mandla	MP	LE
88.	MTCC-6100	*Eutectona machaeralis*	*Lepidoptera*	Mandla	MP	LE
89.	MTCC-6286	*Eutectona machaeralis*	*Lepidoptera*	Mandla	MP	LE
90.	MTCC-6287	*Eutectona machaeralis*	*Lepidoptera*	Mandla	MP	LE
91.	MTCC-6288	*Eutectona machaeralis*	*Lepidoptera*	Mandla	MP	LE
92.	MTCC-6289	*Eutectona machaeralis*	*Lepidoptera*	Mandla	MP	LE
93.	MTCC-6291	*Hyblaea puera*	*Lepidoptera*	Dantewada	CG	LE
94.	MTCC-6297	*Hyblaea puera*	*Lepidoptera*	Dantewada	CG	LE
95.	MTCC-6298	*Eutectona machaeralis*	*Lepidoptera*	Bastar	CG	LE
96.	MTCC-6685	Soil	NA	Dindigul	TN	OT

97.	MTCC-6779	Field infected larva	NA	Dindigul	TN	OT
98.	MTCC-7689	Field infected larva	NA	Dindigul	TN	OT
99.	MTCC-7690	Soil	NA	Thadamparai	TN	OT
100.	MTCC-8017	Silkworm- Bombyx mori	*Lepidoptera*	Rudraprayag	UK	LE
101.	MTCC-9348	Infected *Ideoscopus clypealis*	*Hemiptera*	US Nagar	UK	HE
102.	MTCC-9968	Mango mealy bug	*Hemiptera*	Pant Nagar	UK	HE

HR = Haryana, CG = Chhattisgarh, HP = Himachal Pradesh, MP = Madhya Pradesh, UK = Uttarakhand, TN = Tamil Nadu, NA = not available

Supplementary Table S2: Mycelial growth, sporulation, corrected mortality and bioefficacy index of different entomopathogenic fungi against whitefly (2nd and 3rd instar nymphs)

S.No.	Accession number	Mycelial growth cm	Spore /microgm/ml x 10^8	Corrected mortality (%)			Bioefficacy index (BI)@	BI Reactions#
				3 DAI	5 DAI	7 DAI		
1.	MTCC-2028	23.0	14.5	28.6	50.3	52.0	36.4	MV
2.	MTCC-3653	34.2	0.5	40.8	43.5	46.6	36.0	MV
3.	MTCC-4105	36.5	2.5	11.8	29.2	44.0	35.9	MV
4.	MTCC-4106	11.9	2.8	1.6	3.7	13.2	11.4	PV
5.	MTCC-4107	20.2	1.2	25.0	43.3	45.0	30.1	MV
6.	MTCC-4108	24.0	0.3	15.8	37.2	44.9	31.4	MV
7.	MTCC-4109	18.9	0.7	17.6	59.0	64.3	39.2	MV
8.	MTCC-4110	19.7	8.3	22.7	24.2	25.3	21.1	LV
9.	MTCC-4111	68.9	0.4	32.9	64.8	68.9	60.0	HV
10.	MTCC-4119	40.3	2.5	32.1	34.8	39.8	35.1	MV
11.	MTCC-4120	39.1	2.0	52.6	58.7	60.1	44.8	V
12.	MTCC-4121	27.7	16.7	22.1	27.5	30.8	27.8	LV
13.	MTCC-4122	24.0	6.7	9.5	11.8	16.6	18.0	PV
14.	MTCC-4492	21.6	8.0	16.8	20.1	23.5	20.8	LV
15.	MTCC-4495	32.5	6.2	31.6	41.4	46.3	35.9	MV
16.	MTCC-4496	29.7	2.5	32.6	38.6	49.5	36.0	MV
17.	MTCC-4497	33.1	2.0	28.7	32.1	38.5	31.7	MV
18.	MTCC-4498	24.4	1.7	20.8	23.6	24.1	21.3	LV
19.	MTCC-4499	12.4	3.3	14.5	45.0	52.9	31.5	MV
20.	MTCC-4500	19.3	3.3	18.9	22.3	30.6	22.9	LV
21.	MTCC-4501	37.8	14.3	14.8	16.1	20.9	26.3	LV
22.	MTCC-4503	27.0	2.5	23.1	25.4	32.0	26.3	LV
23.	MTCC-4504	14.4	1.1	31.8	45.0	49.6	30.3	MV
24.	MTCC-4505	21.1	10.3	22.0	24.9	26.4	22.4	LV
25.	MTCC-4506	24.0	1.3	10.7	38.7	59.6	38.9	MV
26.	MTCC-4507	34.0	1.1	12.9	15.4	20.8	23.1	LV
27.	MTCC-4508	18.9	15.7	12.8	56.0	58.5	38.3	MV
28.	MTCC-4510	52.6	1.5	32.9	54.9	57.7	48.5	V

29.	MTCC-4511	59.3	6.5	75.0	88.6	95.1	70.3	HV
30.	MTCC-4512	11.9	2.3	38.1	39.2	44.3	26.9	LV
31.	MTCC-4513	34.3	1.5	68.5	74.1	76.6	51.2	GV
32.	MTCC-4514	18.1	2.0	23.1	29.5	39.9	26.9	LV
33.	MTCC-4515	12.9	2.5	33.8	30.1	40.0	25.1	LV
34.	MTCC-4516	16.4	5.0	31.3	35.9	39.4	26.4	LV
35.	MTCC-4517	69.5	6.8	14.9	24.9	34.9	44.0	V
36.	MTCC-4528	12.6	2.3	45.5	68.5	73.5	41.7	V
37.	MTCC-4530	8.4	1.5	17.0	13.6	20.7	13.7	PV
38.	MTCC-4531	48.8	20.0	21.8	31.4	33.7	37.5	MV
39.	MTCC-4532	21.6	26.5	63.3	73.7	77.5	50.2	GV
40.	MTCC-4533	35.4	3.2	31.4	46.5	71.9	49.5	V
41.	MTCC-4534	44.2	1.3	49.7	64.7	65.7	49.4	V
42.	MTCC-4535	10.5	2.5	76.9	80.0	81.5	45.0	V
43.	MTCC-4536	27.5	3.2	54.2	60.0	69.5	45.3	V
44.	MTCC-4537	37.7	9.9	83.6	83.7	86.4	58.4	GV
45.	MTCC-4538	24.0	0.5	45.0	65.1	68.2	43.1	V
46.	MTCC-4539	24.4	3.3	18.8	64.9	68.2	43.6	V
47.	MTCC-4540	33.0	1.3	18.6	25.5	32.4	28.6	LV
48.	MTCC-4542	16.0	19.1	70.4	72.9	82.8	49.8	V
49.	MTCC-4543	50.3	5.9	67.4	80.0	85.4	62.1	HV
50.	MTCC-4544	11.9	3.2	33.2	48.3	52.8	31.2	MV
51.	MTCC-4546	25.5	3.2	16.9	20.1	22.6	21.1	LV
52.	MTCC-4547	23.5	0.7	13.0	14.3	17.2	17.4	PV
53.	MTCC-4548	49.7	7.8	16.5	23.3	26.0	32.4	MV
54.	MTCC-4549	44.2	2.3	48.3	62.8	73.5	53.4	GV
55.	MTCC-4550	58.3	2.3	27.3	31.1	34.9	39.3	MV
56.	MTCC-4551	20.2	1.8	35.8	37.7	42.5	29.0	LV
57.	MTCC-4552	16.8	1.5	10.7	38.5	40.7	26.8	LV
58.	MTCC-4553	11.2	6.7	14.7	54.7	65.2	37.6	MV
59.	MTCC-4554	56.2	2.0	40.7	52.3	56.5	49.3	V
60.	MTCC-4557	22.0	7.3	62.6	63.4	74.8	46.5	V
61.	MTCC-4559	34.8	2.3	16.0	19.4	28.8	27.6	LV

62.	MTCC-4560	35.3	0.5	35.8	43.0	46.6	36.4	MV
63.	MTCC-4562	28.6	36.8	11.5	13.0	14.7	22.7	LV
64.	MTCC-4563	28.6	1.5	43.4	48.5	49.5	35.5	MV
65.	MTCC-4564	22.1	1.1	30.4	48.4	72.3	44.5	V
66.	MTCC-4565	62.4	1.5	57.2	76.6	89.9	68.2	HV
67.	MTCC-4566	24.8	0.5	59.5	62.5	63.8	41.1	V
68.	MTCC-4567	29.2	2.5	10.2	24.2	27.2	24.7	LV
69.	MTCC-4568	57.7	0.4	58.8	64.5	68.7	55.8	GV
70.	MTCC-4569	18.9	1.2	0.9	3.5	4.7	9.5	PV
71.	MTCC-4571	24.6	1.3	35.3	41.1	42.4	30.5	MV
72.	MTCC-4572	48.2	6.7	37.8	66.3	71.6	54.5	GV
73.	MTCC-4575	49.0	12.8	56.8	69.8	70.3	55.0	GV
74.	MTCC-4576	16.8	16.3	31.3	50.4	52.3	34.5	MV
75.	MTCC-4577	16.8	0.5	39.7	47.5	53.3	33.0	MV
76.	MTCC-4578	13.9	2.0	12.5	14.5	16.6	13.7	PV
77.	MTCC-4579	27.6	0.5	16.1	20.0	25.4	22.9	LV
78.	MTCC-4580	25.2	0.5	17.3	62.8	69.9	44.3	V
79.	MTCC-4581	44.2	1.3	60.0	74.8	78.8	55.9	GV
80.	MTCC-4582	46.2	8.8	20.8	30.4	67.5	52.0	GV
81.	MTCC-4598	11.9	4.2	55.3	56.4	57.7	33.8	MV
82.	MTCC-4599	26.5	2.5	36.6	58.0	59.9	40.1	V
83.	MTCC-4600	25.0	0.5	31.0	33.4	39.6	29.1	LV
84.	MTCC-4605	45.6	0.7	5.4	11.0	12.4	23.2	LV
85.	MTCC-6097	51.7	4.4	23.6	64.4	81.7	60.5	HV
86.	MTCC-6098	27.5	1.7	74.8	73.8	78.7	49.8	V
87.	MTCC-6099	50.3	4.7	46.4	49.5	50.8	44.6	V
88.	MTCC-6100	22.5	1.3	2.9	7.0	11.8	14.4	PV
89.	MTCC-6286	33.1	55.7	39.2	50.6	53.3	46.1	V
90.	MTCC-6287	44.9	3.3	35.5	44.9	45.1	39.6	MV
91.	MTCC-6288	27.6	18.4	82.0	82.6	86.2	55.7	GV
92.	MTCC-6289	16.8	7.5	83.2	87.1	93.8	54.1	GV
93.	MTCC-6291	27.5	3.3	30.8	66.9	72.6	46.9	V
94.	MTCC-6297	27.5	12.7	30.9	48.5	60.5	42.1	V

95.	MTCC-6298	10.5	6.2	18.6	20.1	23.6	16.5	PV
96.	MTCC-6685	35.3	1.3	46.5	47.9	50.7	38.6	MV
97.	MTCC-6779	41.6	1.5	26.5	29.3	24.9	28.0	LV
98.	MTCC-7689	49.0	5.7	5.9	6.9	12.6	25.2	LV
99.	MTCC-7690	24.0	4.3	30.7	43.6	55.6	37.3	MV
100.	MTCC-8017	19.4	1.5	1.7	5.9	16.0	15.4	PV
101.	MTCC-9348	38.4	3.3	46.9	49.6	56.2	42.7	V
102.	MTCC-9968	29.7	3.3	24.3	39.1	46.3	34.6	MV
	LSD at 0.05	8.03	1.61	7.47	7.20	7.84	5.46	
	SE(m)	2.89	0.58	2.68	2.58	2.80	1.97	
	SE(d)	4.08	0.89	3.68	3.65	3.97	2.79	
	CV	15.73	15.74	11.79	9.71	9.61	9.47	

[@]Biological efficacy index (BI) = mycelia growth (mm), sporulation (conidia 1 x 10^8); nymphal mortality 7 days after inoculation (DAI) (%); BI= 0.37 x MG + 0.13 x SP + 0.50 x MO (Sain et al. 2021; Sain et al. 2022)

[#] Reaction: HV = Highly virulent (53.4-70.3); GV= Good virulent (50-59), V= virulent (40-49), MV= moderately virulent (30-39), LV=Low virulent (20-29), PV= poor virulent (below 19)
